# Can the collective intentions of individual professionals within healthcare teams predict the team's performance: developing methods and theory

**DOI:** 10.1186/1748-5908-4-24

**Published:** 2009-05-05

**Authors:** Martin P Eccles, Susan Hrisos, Jillian J Francis, Nick Steen, Marije Bosch, Marie Johnston

**Affiliations:** 1 Institute of Health and Society, University of Newcastle upon Tyne, 21 Claremont Place, Newcastle upon Tyne, NE2 4AA, UK; 2 Health Services Research Unit, University of Aberdeen, 3rd Floor, Health Sciences Building, Foresterhill, Aberdeen, AB25 2ZD, UK; 3 School of Psychology, William Guild Building, University of Aberdeen, Aberdeen, AB24 2UB, UK; 4 Scientific Institute for Quality of Healthcare, Radboud University Nijmegen Medical Centre, Radboud University Nijmegen, Geert Grooteplein 21, 6525 EZ, Nijmegen, The Netherlands

## Abstract

**Background:**

Within implementation research, using theory-based approaches to understanding the behaviours of healthcare professionals and the quality of care that they reflect and designing interventions to change them is being promoted. However, such approaches lead to a new range of methodological and theoretical challenges pre-eminent among which are how to appropriately relate predictors of individual's behaviour to measures of the behaviour of healthcare professionals. The aim of this study was to explore the relationship between the theory of planned behaviour proximal predictors of behaviour (intention and perceived behavioural control, or PBC) and practice level behaviour. This was done in the context of two clinical behaviours – statin prescription and foot examination – in the management of patients with diabetes mellitus in primary care. Scores for the predictor variables were aggregated over healthcare professionals using four methods: simple mean of all primary care team members' intention scores; highest intention score combined with PBC of the highest intender in the team; highest intention score combined with the highest PBC score in the team; the scores (on both constructs) of the team member identified as having primary responsibility for the clinical behaviour.

**Methods:**

Scores on theory-based cognitive variables were collected by postal questionnaire survey from a sample of primary care doctors and nurses from northeast England and the Netherlands. Data on two clinical behaviours were patient reported, and collected by postal questionnaire survey. Planned analyses explored the predictive value of various aggregations of intention and PBC in explaining variance in the behavioural data.

**Results:**

Across the two countries and two behaviours, responses were received from 37 to 78% of healthcare professionals in 57 to 93% practices; 51% (UK) and 69% (Netherlands) of patients surveyed responded. None of the aggregations of cognitions predicted statin prescription. The highest intention in the team (irrespective of PBC) was a significant predictor of foot examination.

**Conclusion:**

These approaches to aggregating individually-administered measures may be a methodological advance of theoretical importance. Using simple means of individual-level measures to explain team-level behaviours is neither theoretically plausible nor empirically supported; the highest intention was both predictive and plausible. In studies aiming to understand the behaviours of teams of healthcare professionals in managing chronic diseases, some sort of aggregation of measures from individuals is necessary. This is not simply a methodological point, but a necessary step in advancing the theoretical and practical understanding of the processes that lead to implementation of clinical behaviours within healthcare teams.

## Background

Within implementation research – the scientific study of methods to promote the uptake of research findings, and hence to reduce inappropriate care – using theory-based approaches to understanding the behaviours of healthcare professionals and the quality of care that they reflect and designing interventions to change them is being promoted [1,2]. However, such approaches lead to a new range of methodological and theoretical challenges pre-eminent among which are how to appropriately relate predictors of individual's behaviour to measures of the behaviour of healthcare professionals [3]. Commonly (at least within the UK and the Netherlands), data on the quality of care that patients receive within a primary care practice will indicate that various clinical behaviours have been performed, but it may not be possible to identify which individual healthcare professional (HCP) within the clinical team uniquely performed them, or the data may be a reflection of the actions of more than one individual healthcare professional.

While it is possible, and in certain circumstances appropriate and feasible, to directly observe the behaviour(s) of HCPs this is likely to be expensive, time consuming, and ethically problematic. In studies concerned with improving the quality of care that patients receive, it is more commonly the case that various forms of routinely available data are used. Such data that represent a proxy, or indirect, measure of HCP behaviour usually fall into two categories; recorded measures of HCP behaviour (*e.g*., prescription of a statin, reflecting behaviour in relation to the management of hypercholesterolaemia) and clinical, physiological, or biochemical measures of the patient's condition (*e.g*., serum cholesterol level). However, prescriptions apparently issued in the name of one doctor may have actually been issued by trainee doctors or locums. In addition, the prescribed treatment of an individual patient may be changed by different doctors over time. Similarly, a measure of a patient's serum cholesterol may also reflect the behaviours of more than one HCP – a nurse may advise a patient about their diet and a doctor may prescribe a statin. Such considerations apply to any chronic condition managed by a team of healthcare professionals in primary care, *e.g*., diabetes, heart disease, asthma, or chronic obstructive airways disease. Such data are most appropriately considered as practice-level data. However, measurement of factors aimed at improving practice-level quality of care through changing the behaviour of HCPs often occurs at an individual level. It is therefore important to develop methods of predicting clinical behaviours that can take account of the collective performance of individuals working in teams.

### Theoretical context

Explanations for clinical behaviour can be investigated using psychological theories which have been successful in predicting behaviour and behaviour change in other settings. Using such a theory-based approach offers the potential of a generalisable framework within which to consider factors influencing behaviour and the development of interventions to modify them. A study by Eccles *et al*. [3] used six theories to investigate factors associated with prescribing antibiotics for patients with a sore throat among primary care doctors. This showed that the impact of individual beliefs and perceptions on intention to prescribe was high, including both evidence-based and non-evidence based factors, while the impact on behaviour was considerably smaller. Two systematic reviews of the relationship between intention and behaviour in individual HCPs [4,5] found only 16 eligible studies but suggested that the nature of the relationship was similar to that shown by reviews of much larger numbers of studies in non-healthcare professionals [6]. Data such as these allow clear predictions to be made about the factors likely to change psychological constructs and to change behaviour.

**Figure 1:**
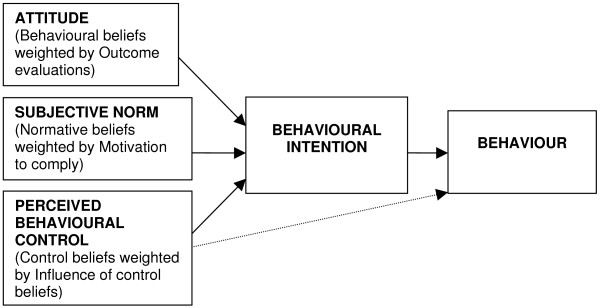
**The Theory of Planned Behaviour **[7]. (Note. The three proximal variables also influence one another. Although this figure is presented in a simplified form, a more detailed diagram would include double-ended arrows joining these three variables.)

One of the more widely used theories is the theory of planned behaviour (TPB) [7]. The TPB proposes a model about how human action is guided. It predicts the occurrence of a specific behaviour provided that the behaviour is intentional (i.e. the model does not claim to predict behaviours that are habitual or automatic). The TPB model is shown in Figure 1 and depicts the three cognitive variables that the theory suggests will predict the intention to perform a behaviour. While intention is the main precursor of behaviour, perceived behavioural control (PBC) also directly predicts behaviour. For example, a positive intention may be prevented from being translated into action because of an internal or external barrier that the individual perceives as insurmountable.

Because data may reflect the behaviour of more than one HCP, it is thus appropriate to analyse these proxy behaviour data at the aggregated level of a primary care practice. Thus, recorded data can indicate reliably that a patient has been prescribed a statin by one of the HCPs in the practice. However, in order to use a theory-based approach it is then necessary to also consider aggregating individuals' measurement of cognitions (about the prescribing of statins). It would be possible to aggregate measures of individuals' cognitions about clinical behaviours and conditions as a simple mean (as is the practice in the literature on measurement of team-level variables such as team climate[8]). However, the mean may not reflect the organisational, professional, and social processes involved in the team. It may be possible to improve the predictive performance of measures that represent team cognitions by taking account of factors such as individuals' roles, responsibilities, or positions. For example, when identified individuals predominantly perform, or have responsibility for, a behaviour (foot examination of patients with diabetes by a practice nurse), then that individual's intention score could be used as a sole representative measure or used to weight a mean value.

### Clinical context

Type 2 diabetes mellitus (DM) is an increasingly prevalent chronic illness and is an important cause of avoidable mortality. Studies of the quality of care for patients with diabetes suggest less than optimum care in a number of areas [9]. In primary care, the management of DM includes glycaemic control, blood pressure control, foot examination for peripheral pulses and neuropathy, lipid control, and weight reduction (retinopathy screening is often organised separately from the practice). Patients are managed by the integrated activities of medical and non-medical members of the primary care team.

### Aim

The aim of this study was to explore the relationship between the TPB's direct predictors of behaviour (individuals' intention and PBC) aggregated over HCPs in a number of ways, and practice level behaviour in the context of care for patients with DM in primary care.

The method of aggregation is not simply a statistical device but may reflect different team processes and different theoretical approaches to team-functioning. For example, aggregating intentions by averaging suggests equal weighting of members' views and would suggest team decision-making based on equal and shared communications. Whereas, choosing the highest intention score in the team to represent the relevant 'team cognition score', suggests that the team has allocated roles, with one member specialising in, or having responsibility for, the targeted clinical behaviour; here the underlying model suggests a more complex team structure with more streamlined decision-making. Other methods of aggregating would also test specific role structures, *e.g*., the team process may be best assessed by selecting the highest intention indicating responsibility for decision-making, along with highest PBC indicating responsibility and capability for the actual behaviour.

Therefore, we investigated the following methods of aggregating respondents' scores for each primary care team: simple mean of all PC team members' intention scores; highest intention score from responding HCPs combined with PBC for either that individual, or, for the highest PBC, the scores of the HCP identified as having primary responsibility for the clinical behaviour, ignoring the scores of other team members.

## Methods

### Design and participants

This was a predictive study of the theory-based cognitions and clinical behaviours concerning the management of patients with diabetes of a sample of primary care doctors and nurses from northeast England, and primary care doctors, nurses, and practice assistants in the Netherlands. We regarded all the healthcare workers within a practice as a team. Data on roles and cognitions were collected by postal questionnaire survey; behavioural data were patient-reported and collected by postal questionnaire survey. Planned analyses explored the predictive value of various aggregations of intention and PBC in explaining variance in the behavioural data.

### Study setting

The study was based within two randomised controlled trials of interventions to improve the management of patients with diabetes cared for in primary care.

### Study practices

In the UK, the study practices were those in three primary care trusts (PCTs) served by two district hospital-based diabetes registers both using the same register software [10]. In the Netherlands, the practices were those in three regions of the middle and south of the Netherlands [11].

### Study patients

In the UK, the study patients were those people with type 2 diabetes appearing on the area-wide diabetes registers, aged over 35 and receiving diabetes care exclusively from the DREAM trial (The **D**iabetes **RE**call **A**nd **M**anagement system trial) [10] practices, or shared between study practices and hospital. At the time of the study, approximately 20% of patients received both general practitioner (GP) and specialist care, though there was no formal shared-care scheme in operation in the practices studied. In the Netherlands, patient reported outcomes were gathered from patients with type 2 diabetes, who were younger than 80 years and registered with practices participating in the PAS trial (The diabetes **P**assport as an **A**id to **S**tructure diabetes management in primary care trial) [11]. Patients managed in secondary care were excluded from the PAS trial.

### Predictive measures

Theoretically-derived measures were developed following the operationalisation protocols of Ajzen [7,12]. Twelve UK primary care doctors and practice nurses were interviewed about three behaviours (measuring blood pressure, foot examination, prescribing statins). The schedule for these semi-structured interviews was designed to elicit responders' beliefs relating to the constructs of the TPB. Primary care doctors and practice nurses were encouraged to talk freely about these beliefs, and any ambiguities were clarified using appropriate prompts. Interviews were tape recorded, transcribed, and content analysed. Beliefs frequently mentioned in the interviews were used to design items in a questionnaire that was developed for each of the three behaviours. The response format for all items was a seven point Likert-type scale, from one (strongly agree) to seven (strongly disagree). This initial draft of the questionnaire was pre-tested with a further six UK primary care doctors for style and clarity of content and to determine completion time. Minor revisions of wording were made to the questionnaire based on their comments. The final questionnaire used in the UK covered three behaviours, both 'indirect' and 'direct' measures of the theoretical constructs [7,12] and consisted of 154 items, including questions about the size of practices and demographic details. For the Netherlands survey, because of concerns about respondent burden, a shortened set of the questions from the UK questionnaire was used covering only two of the three behaviours and using only direct measures. The relevant questions from the UK set were translated into Dutch and then back translated into English (and adjusted where necessary) to ensure that the meaning was the same for the UK and Dutch studies.

The questions measuring intention and PBC for the two behaviours of prescribing statins and examining patients' feet are shown in the Appendix. Scoring was adjusted so that a high score indicates a strong intention and a high degree of perceived control.

### Outcome measures

In the UK, as part of a larger patient reported outcomes survey [10], patients with DM were asked the following two questions. First, 'please provide as much information as you can in the box below about **ALL **the medication you have taken **over the last four weeks **'; any report of a statin was identified. Second, they were asked, 'over the last 12 months did you have any of the tests or investigations listed'; the list included: 'test of feeling on your feet'; a positive response was taken as an indication of having a foot examination.

In the Netherlands, patients were asked to report on the medication they were currently taking and whether or not they had had their feet examined in the past 15 months.

For both countries, responses were used to calculate the percentage of patients per practice who reported taking a statin, and the percentage of patients per practice who reported having their feet examined.

### Procedure

In both the UK and the Netherlands, the questionnaire was mailed to all primary care doctors, nurses, and (in the Dutch practices) practice assistants at participating trial practices at the end of the intervention period. In the UK, two reminder letters were sent to non-responders at fortnightly intervals. Dutch non-responders received one reminder letter after three weeks. Patient reported outcomes were also collected by postal questionnaire at the end of the intervention period of both trials.

### Analytical approach

Internal consistency of multi-item measures [of intention and PBC] was assessed using Cronbach's alpha (for measures with more than two items) using an acceptability criterion of α >0.6, and Pearson's correlation coefficient (for two-item measures) using an acceptability criterion of r >0.25.

We were interested in the relationship between practice-level behaviour and aggregations of individuals' cognitions (intentions and PBC), and investigated this using multiple regression analysis. We conducted analyses to reflect four possible team patterns. First, we argued that the behaviour was likely to be driven equally by the individual intentions of all the practice members; we therefore calculated a mean value for each practice. It was likely that we would both get responses from single-doctor practices and get single responses (from either a nurse or a doctor) from multi-doctor practices. Under these circumstances the concept of a mean value was less meaningful, and therefore we repeated the analyses including only those practices from which we received more than one response. Second, we considered that behaviour could be most driven by the individual with the highest intention (and their PBC) within the practice, and so used these measures as predictor variables. Third, we considered that the behaviour could be the product of one team member having a strong intention, and another team member having a high level of PBC. An example of this would be the situation where a nurse had a high intention to perform the behaviour and a doctor had a high PBC score as a consequence of knowing that the nurse intended to perform the behaviour. Fourth, we considered that behaviour was most likely to be driven by the individual whose role it was to perform the behaviour. Therefore, for foot examination, we considered that this could be the role of a nurse. The statin analysis was restricted to doctors.

As the TPB predicts a direct effect of both intention and PBC on behaviour, both were included in the regression analyses.

We also explored a country effect (to allow for both 'real' and methodological differences between them) and the number of responses per practice. Although both host studies were randomised controlled trials, we analysed them as two cross-sectional studies on the basis that any effect of the interventions on behaviour would be mirrored by a change in cognitions, and that the relationship between cognitions and behaviour should therefore persist, whether or not the trial changed the levels observed in the intervention group.

### Ethical approval

The UK study was approved by the South Tyneside, Southwest Durham, Hartlepool, and North Tees Local Research Ethics Committees (LRECs). The Dutch study was approved by the ethics committee of Radboud University Medical centre, Nijmegen, The Netherlands.

## Results

The details of the number of healthcare professionals surveyed and the characteristics of their practices, as well as the survey response rates are shown in Table 1. Overall, 98 practices were surveyed and health professionals from 83 (85%) practices returned questionnaires. Practices were dichotomised into single- or multi-practitioner practices. Of the 83 practices, the 69 contributing at least one GP responder to the statin analysis were not significantly different in terms of size to non-responder practices (Pearson χ^2 ^= 2.248, d.f. = 1, p = 0.13). For the analysis of foot examination, the number of nurses per practice was also available. In the Dutch study, this included eight nurses and 14 assistants who inspected feet, and excluded 26 assistants who did not inspect feet.

**Table 1:** Characteristics of sample and questionnaire response rates from healthcare professionals for the two behaviours.

	**Overall**			**Response rates (n (%))**					
	**Numbers**			**Statin prescription**			**Foot examination**		

	**UK**	**Dutch**	**Total**	**UK**	**Dutch**	**Total**	**UK**	**Dutch**	**Total**

**Number of HCPs**									

**primary care doctors**	161	59	220	59 (37)	46 (78)	105 (48)	59 (37)	46 (78)	105 (48)

**Nurses**	119	22*	141				53 (45)	19 (86)	72 (51)

**Practices**									

**Overall**	58	40	98	34 (57)	35 (88)	69 (70)	46 (79)	37 (93)	83 (85)

**Single primary care doctor**	15	15	30	7 (21)	11 (31)	18 (26)	10 (22)	13 (35)	23 (28)

**>1 primary care doctor**	43	25	68	27 (79)	24 (69)	51 (74)	36 (78)	24 (65)	60 (72)

**Number (Median (range))/practice**									

**primary care doctors**	2 (1–9)	2 (1–4)	2 (1–9)	3 (1–9)	2 (1–4)	2 (1–9)	3 (1–9)	2 (1–4)	2 (1–6)

**Nurses**	2 (1–6)	2 (1–5)	2 (1–6)	1 (1–6)	1 (1–2)	1 (1–6)	2 (1–4)	2 (1–5)	1 (0–6)

*Includes eight nurses and 14 assistants who inspect feet; excludes 26 assistants who did not inspect feet.

Practices were again dichotomised, and the 83 practices contributing at least one responder to this analysis were not significantly different in terms of the number of primary care doctors in the practice (Pearson χ^2 ^= 2.149, d.f. = 1, p = 0.14); but were significantly more likely to have two or more nurses (80% versus 47%, Pearson χ^2 ^= 7.215, d.f. = 1, p = 0.007).

In the UK study, a random sample of 2,815 patients were surveyed, and usable responses were received from 1,433 (51%). In the Dutch study, 1,432 patients were surveyed, with 993 (69%) usable responses received. Overall, 736/2,426 (30%) patients reported taking statins (362/1,433 (25%) UK patients and 374/993 (38%) Dutch patients). Overall, 1,234/2,426 (51%) patients reported having their feet examined in the past 12 (UK) or 15 (Dutch) months (806/1,395 (58%) UK patients and 428/993 (43%) Dutch patients).

### Prescribing statins

The three-item measure of intention had a Cronbach's alpha of 0.95. The two item measure of PBC had a Pearson's Correlation Coefficient of 0.37 (p < 0.001). In UK practices, the overall mean (sd) of the practice mean intention score was 4.8 (1.5), and in Dutch practices this was 5.6 (1.3) (mean difference (95% CI) -0.7300 (-1.4 to -0.04) p = 0.038). Similar values for the strongest intention were, for the UK practices, 5.2 (1.5) and for the Dutch practices 5.7 (1.3); these were not significantly different. The mean intention score (from participating HCPs) within each practice was significantly correlated with the highest intention score within that practice (Pearson Correlation Coefficient 0.93, p < 0.001), but neither was significantly correlated with the practice mean percentage of patients taking a statin.

In a regression model including both mean intention and mean PBC (Table 2), neither significantly predicted behaviour but there was a significant 'country effect' with Dutch primary care doctors being 11% more likely to prescribe statins. When PBC was removed from the model, intention still did not predict behaviour and there was no additional effect of an interaction term between intention and country (*i.e*., intention was not a significantly greater predictor in one country than the other). A similar analysis restricted to the smaller number of practices where there was more than one respondent produced a similar pattern of results, though the country effect was not significant.

**Table 2:** Regression models for mean and strongest intention for statin use and foot examination.

**Model**	**n**	**Predictive Variables**	**B**	**Beta**	**R ^**2 **^(adj)**	**F**	**p value**
**Prescribing statins**							

Mean intention (all practices)	69	Mean intention	0.005	0.05			
		Mean PBC	-0.006	-0.034			
		Country	0.11	0.389**	0.127	4.312	0.008

Mean intention (practices with >1 respondent)	25	Mean intention	-0.003	-0.036			
		Mean PBC	0.044	0.237			
		Country	0.093	0.350	0.03	1.239	0.321

Highest	69	Highest intention	0.001	0.011			
Intention (a)		PBC of strongest intender	0.016	0.108			
		Country	0.115	0.406***	0.136	4.560	0.006

Highest	69	Highest intention	0.003	0.027			
Intention (b)		Highest PBC	0.001	0.006			
		Country	0.113	0.401***	0.125	4.244	0.008

**Foot examination**							

Mean intention (all practices)	83	Mean intention	-0.003	-0.017			
		Mean PBC	-0.016	-0.084			
		Country	-0.125	-0.322**	0.097	3.922	0.012

Mean intention (practices with >1 respondent)	51	Mean intention	-0.001	-0.006			
		Mean PBC	0.000	0.000			
		Country	-0.075	-0.224	-0.11	0.826	0.486

Highest	83	Highest intention	0.033	0.229*			
Intention (a)		PBC of Highest intender	-0.008	-0.054			
		Country	-0.113	-0.289*	0.138	5.390	0.002

Highest	83	Highest intention	0.034	0.239*			
Intention (b)		Highest PBC	-0.008	-0.048			
		Country	-0.116	-0.297**	0.138	5.363	0.002

*p < 0.05, **p < 0.01, ***p < 0.001

When using the highest intention score for each practice, none of highest intention, PBC of the highest intender, or highest PBC in the practice predicted the prescription of statins (Table 2). Again, the country effect is apparent and of the same order of magnitude and significance. When PBC was removed from the model, intention still did not predict behaviour, and there was no additional effect of an interaction term between intention and country.

### Foot examination

The three-item measure of intention had a Cronbach's alpha of 0.96. The two-item measure of PBC had a Pearson's Correlation Coefficient of 0.44 (p < 0.001). In UK practices, the overall mean (sd) of the practice mean intention score was 4.9 (1.3), and in Dutch practices this was 4.4 (1.4); these were not significantly different. Similar values for the strongest practice intention were, for the UK practices, 5.9 (1.3) and for the Dutch practices 5.1 (1.6) (Mean difference (95%CI) 0.78 (0.14 to 1.43), p = 0.018). The mean intention score for a practice was significantly correlated with the highest intention score within that practice (Pearson Correlation Coefficient 0.78, p < 0.01) and the highest intention score was also significantly correlated with the practice mean percentage of patients reporting a foot examination (Pearson Correlation Coefficient 0.29, p < 0.01).

In a regression model (Table 2) including both mean intention and mean PBC, neither significantly predicted behaviour but there was a significant 'country effect' with UK practices being 14% more likely to inspect feet. When PBC was removed from the model, intention still did not predict behaviour, and there was no additional effect of an interaction term between intention and country. A similar analysis restricted to the smaller number of practices where there was more than one respondent produced a similar pattern of results, though the country effect was not significant.

The highest intention score in a practice belonged to 38 nurses (24 of whom were from practices where intention scores were available for both primary care doctor and nurse respondents) and 39 primary care doctors (eight of whom were from practices where intention scores were available for both primary care doctor and nurse respondents). In the remaining six practices, this score was the same for both nurse and primary care doctor, and the regression used the scores for individuals who have both the highest intention and the highest PBC. The highest practice intention was a significant predictor of foot examination. Again, there was a significant country effect, with reported feet inspections being 11% fewer in ND practices than UK practices (p = 0.011). Removing PBC, including an interaction term for intention/country and including type of healthcare professional (thus exploring professional role) did not significantly change the model. Finally, the analysis was repeated using the highest intention score for the practice and the strongest PBC score for the practice. In this model, the PBC score is predominantly that of the primary care doctor respondents. This analysis produced results similar to the previous one.

## Discussion

This paper reports an analysis of four different ways of dealing with the problem of relating the cognitions of individual members of a team of healthcare professionals to a shared outcome of their collective behaviours. For the behaviour of foot examination, how the individual cognitions were analysed made a difference with strongest intention, not mean intention, being significantly associated with practice level behaviour. However, this has to be regarded as exploratory and preliminary in a number of ways.

The theories we were using were not necessarily intended to be used as we have used them, and we are proposing an extension of the use of the TPB to the collective behaviour of a team. Pragmatically, there does not seem to be any reason why measures cannot be used in this way. Indeed, other measures of team performance, such as the team climate inventory, use a simple mean as their summary statistic [8]. In a theoretical context, it is unclear what a team's mean intention score represents. However, as suggested earlier, if mean intention is predictive, it suggests some kinds of collective processes, especially with regard to decision-making and communication. Our finding that mean intention was not predictive (while acknowledging our limited numbers and response rates), suggests that for the management of these two clinical behaviours by primary care teams, decision-making and responsibility may not be distributed equally across the team.

We were using a cognitive model for what seem to be intentional behaviours. However, these are relatively routine behaviours and they may well, over time, become routinely maintained and therefore no longer need thinking through each time they are performed. Therefore other measures, either instead of or alongside social cognition models, may have additional predictive power for teams. Indeed, in a study of primary care practitioners' antibiotic prescribing behaviour that compared the predictive power of theories, a measure of habit was the best predictor of behaviour [3].

While mean levels of intention to perform both behaviours were positive, being between 4.4 and 5.6 for both behaviours in both countries, levels of performance for what should be almost universal behaviours were low; for only foot examination in the UK was the reported rate of performance about 50%. This could be due to: low reporting rates by patients (our source of this data); the potential mismatch for prescribing statins arising from patients reporting what they were taking and doctors reporting their intention to prescribe; or bias (*e.g*., social desirability) in reporting of intention by healthcare professionals. However, it could also indicate the possibility of there being post-intentional factors which we have not measured that are influencing behaviour, such as intention stability, habit, and anticipated regret.

The finding that the strongest intention score within each team, for inspecting feet, significantly predicted patients' reports of foot inspection, is consistent with the possibility that healthcare professionals may have had stronger intentions if they had been assigned responsibility for foot inspection within the practice (though our attempt to allocate roles in our analyses did not confirm this). The idea that assigned roles and responsibilities influence cognitions and behaviour has received substantial support in the behavioural literature [13,14]. An alternative possibility is that teams allocate responsibility for a task to those with the strongest intentions to perform it, *i.e*., that roles evolve and may be chosen rather than being allocated. These possibilities warrant further investigation.

While we explored different ways of relating behaviour and its theorised predictors, our data from patients and healthcare professionals had limitations. The measures of behaviour were collected by patient self-report and so may be subject to recall and other biases. However, these measures were the only measures in common for these behaviours across the two host trials. Encouragingly, the rates of statin use and foot inspection reported by the English patients in this study are supported by additional data from medical records reported elsewhere [10]. This provides a degree of validation that these proxy measures provided a measure near to that of actual rates of statin prescription and foot inspection. In the UK sample, 20% of the patients had their care shared between primary and secondary care. We cannot quantify the impact of this but it should be specifically examined in future work.

We know that across individual practices we usually had only a minority of team members responding so that the team mean scores did not include scores from those disinclined to complete questionnaires. The implication of this is that we may have lacked the power to detect difference across the different analyses. Also, if individual healthcare professionals do have a specified role within a practice (*e.g*., to inspect patients' feet), we do not know whether that individual responded to the questionnaire. If individuals with the highest intention within the team, or with the assigned responsibility, did not respond, then we may have underestimated these effects. While non-response is an enduring issue for health services research in general, an ideal study of this type would include responses from all members of the participating teams.

## Conclusion

However exploratory this work, the issues raised are of enduring importance, both methodologically and theoretically [15]. In studies wishing to understand the behaviours of healthcare professionals in relation to the management of many chronic diseases then some sort of aggregation of measures from individuals is inevitably going to be necessary. Given that so much of healthcare involves teams of healthcare professionals, the issues addressed in this study, however imperfectly, need to be addressed. This is not simply a methodological point but a necessary step in advancing the theoretical and practical understanding of the processes that lead to implementation of clinical behaviours within healthcare teams.

## Competing interests

The authors declare that they have no competing interests.

## Authors' contributions

MPE, MJ and JF conceived the study. MPE, JF, SH and MB were responsible for data collection. MJ and NS supervised the analyses. MPE led the writing and all authors commented on sequential drafts and approved the final version of the manuscript.

## Appendix

**Questions measuring intention and perceived behavioural control for the two clinical behaviours**.

Each question in the following section refers to the PRESCRIBING OF STATINS to your patients with Type 2 diabetes

Intention questions

I intend to prescribe a statin to most of the patients I see in the next month

I expect to prescribe a statin to most of the patients I see in the next month

I want to prescribe a statin to most of the patients I see in the next month

Perceived behavioural control questions

To prescribe a statin is easy

Overall, I feel that I can prescribe statins if I want to

Each of the questions in the following section refers to FOOT EXAMINATIONS on your patients with Type 2 diabetes

Intention questions

I intend to examine the feet of all my patients I see in the next month who have not been examined by the chiropodist or the podiatrist

I expect to examine the feet of all my patients I see in the next month who have not been examined by the chiropodist or the podiatrist

I want to examine the feet of all my patients I see in the next month who have not been examined by the chiropodist or the podiatrist

Perceived behavioural control questions

Examining patients' feet is easy

Overall I feel that I can examine these patients' feet if I want to
